# Pyroptosis, metabolism, and tumor immune microenvironment

**DOI:** 10.1002/ctm2.492

**Published:** 2021-08-03

**Authors:** Tiantian Du, Jie Gao, Peilong Li, Yunshan Wang, Qiuchen Qi, Xiaoyan Liu, Juan Li, Chuanxin Wang, Lutao Du

**Affiliations:** ^1^ Department of Clinical Laboratory The Second Hospital Cheeloo College of Medicine Shandong University Jinan Shandong China; ^2^ Shandong Engineering and Technology Research Center for Tumor Marker Detection Jinan Shandong China; ^3^ Shandong Provincial Clinical Medicine Research Center for Clinical Laboratory Jinan Shandong China

**Keywords:** gasdermin, metabolism, pyroptosis, tumor immunity, tumor microenvironment

## Abstract

In response to a wide range of stimulations, host cells activate pyroptosis, a kind of inflammatory cell death which is provoked by the cytosolic sensing of danger signals and pathogen infection. In manipulating the cleavage of gasdermins (GSDMs), researchers have found that GSDM proteins serve as the real executors and the deterministic players in fate decisions of pyroptotic cells. Whether inflammatory characteristics induced by pyroptosis could cause damage the host or improve immune activity is largely dependent on the context, timing, and response degree. Here, we systematically review current points involved in regulatory mechanisms and the multidimensional roles of pyroptosis in several metabolic diseases and the tumor microenvironment. Targeting pyroptosis may reveal potential therapeutic avenues.

## Graphical abstract

1. Gasdermins are executors of pyroptosis, which cause inflammatory cell death, accompanied with various inflammatory mediators.
2. Pyroptosis plays a dual role in tumor microenvironment, including anti‐tumor and pro‐tumor effects.
3. Pyroptosis can be used as a new target to fight against the tumor.

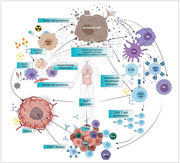

**Abbreviations**
AKT: protein kinase B
ANSD: auditory neuropath spectrum disorder
ASC: apoptosis‐associated speck‐like protein containing a CARD
ATP: adenosine triphosphate
CAR: chimeric antigen receptor
CatG: Cathepsin G
CCA: extrahepatic cholangiocarcinoma
cGAS: cyclic GMP‐AMP synthase
CRC: colorectal cancer
CRS: cytokine‐release syndrome
CT: C‐terminal
DAC: decitabine
DAG: diacylglycerol
DAMPs: damage‐associated molecular patterns
DCs: dendritic cells
DHA: docosahexaenoic acid
DN: diabetic nephropathy
dsDNA: double‐stranded DNA
ECs: endothelial cells
ELANE: neutrophil elastase
ER: endoplasmic reticulum
ERK: extracellular signal‐regulated kinase
ESCRT: endosomal sorting complexes required for transport
GGA: geranylgeranoic acid
GSDMD: gasdemin D
GSDME: gasdemin E
GSDMs: gsdermins
GZMA: granzyme A
GZMB: granzyme B
Hcy: Homocysteine
HER2: human epidermal growth factor receptor 2
HFD: high‐fat diet
HG: high‐glucose
HMGB1: high‐mobility group box 1
HUVECs: human umbilical vein endothelial cells
ICD: inflammatory cell death
ICI: immune checkpoint inhibitor
IFN‐γ: interferon‐gamma
IRF2: IFN regulatory transcription factor‐2
JNK: c‐Jun N‐terminal kinase
LOF: loss‐of‐function
LPC: lysophosphatidylcholine
LPS: lipopolysaccharide
LUAD: lung adenocarcinoma
MDMs: monocyte‐derived macrophages
MLZE: melanoma‐derived leucine zipper extranuclear factor
mtDNA: mitochondrial DNA
NaB: sodium butyrate
NAFLD: non‐alcoholic fatty liver disease
NFATc1: nuclear factor of activated T‑cells, cytoplasmic 1
NF‐κB: nuclear factor kappa B
NK: natural killer
NT: N‐terminal domain
OA: oleic acid
ox‐LDL: oxidized low‐density lipoprotein
P2 × 7R: P2 × 7 receptor
PA: palmitic acid
PD‐L1: programmed death‐ligand 1
PGE2: prostaglandin E2
PRRs: pattern recognition receptors
RIPK1: receptor‐interacting serine/threonine protein kinase 1
ROS: reactive oxygen species
S1P: sphingosine‐1‐phosphate
S1PR2: S1P receptor 2
SGNs: spiral ganglion neurons
SIRT1: Sirtuin1
SM: sphingomyelin
SMSs: sphingomyelin synthases
SSc: systemic sclerosis
STAT3: signal transducer and activator of transcription 3
T1D, T2D: Type 1 and 2 diabetes
T3SS: type‐3 secretion system
TcdB: Clostridium difficile (C. difficile) toxin B
TET2: tet methylcytosine dioxygenase
TILs: tumor‐infiltrating lymphocytes
TLR4: toll‐like receptor 4
TMAO: trimethylamine N‐oxide
TME: tumor microenvironment
TNF‐α: tumor necrosis factor alpha
UQCRC1: ubiquinol cytochrome c reductase core protein I
VSMCs: vascular smooth muscle cells
YAP: yes‐associated protein

## 1 INTRODUCTION

In 1992, the initial phenomenon of pyroptosis was first discovered; this is a novel type of suicide in macrophages infected by *Shigella flexneri*, a type of gram‐negative bacterial pathogen.^1^ Then, in 2001, pro‐inflammatory programmed cell death termed “pyroptosis” was defined, which distinguishes it from apoptosis.^2^ However, the molecular mechanism of pyroptosis is still not clear; caspase‐1 was considered the critical player for a long time until the cleavage of GSDMD was found.^3^ Since then, pyroptosis has been characterized by GSDM‐mediated membrane pore‐formation and cytokine release. Surprisingly, pyroptosis does not always lead to lytic cell death; in some contexts, there is a damage impair system named endosomal sorting complexes required for transport (ESCRT), which could eliminate the pores of gasdermins (GSDMs) and protect the plasma membrane from rupture.^4^ The repair system develops a new understanding for the mechanism of pyroptosis.^5^ With further research into inflammatory cell death (ICD), pyroptosis was shown to be a two‐sided sword in different contexts. On the one hand, moderate pyroptosis facilitates cells to maintain homeostasis, improve immune activity, and effectively clear damage and pathogens to protect the host,^6^, ^7^, ^8^, ^9^ which benefits antitumor immunotherapy and nanodrug researches.^6^, ^10^ On the other hand, excessive inflammation caused by pyroptosis is unfavorable to the host and may aggravate disease development, especially tumor progression, which releases various inflammatory factors and forms an inflammatory immune microenvironment^11^, ^12^ (Table 1).

**TABLE 1:** Major events in the history of pyroptosis

Time	Events	Refs.
1986	Arthur Friedlander showed that anthrax lethal toxin (LT) induced robust cell death with rapid release of cellular contents in primary mouse macrophages	^13^
1989	ICE (interleukin‐1β‐converting enzyme, caspase‐1) was first discovered as a pre‐aspartate‐specific protease by cleaving pro‐interleukin‐1 beta (IL‐1β)	^14^
	ICE was identified as unique cysteine protease to process the IL‐1β precursor into mature IL‐1β.	^15^, ^16^
1992	*Shigella flexneri* infection induced suicide in mouse macrophages, and it was regarded as programmed cell death (apoptosis)	^1^
1996	ICE was activated during *Shigella flexneri* infection by directly binding with IpaB.	^17^
1998	Genetic mutations of *DFNA5* (*GSDME*) linked to non‐syndromic hearing impairment	^18^
2000	*Gasdermin* (*Gsdm*, *Gsdma1*) was first named. And, it was the first report that its expression was restricted to both upper gastrointestinal (GI) tract and skin.	^19^
2001	The term “pyroptosis” (from the Greek roots *pyro*, relating to fire or fever, and *ptosis* (*tosis*) to denote a falling,) was first proposed to describe pro‐inflammatory programmed cell death (*Salmonella*‐induced death).	^2^
2001	*MLZE* (*GSDMC*) as a novel gene was first isolated whose expression in accordance with metastatic ability of melanoma cells.	^20^
2002	The term “inflammasome” was first put forward to replace caspase‐activating complex, which can activate inflammatory caspases and process pro‐IL‐1β.	^21^
2004	*GSDML* (*GSDMB*) was identified as homologous gene cluster of *GSDM* (*GSDMA*) gene.	^22^
	*Gsdm*‐related genes were first designate as *Gsdm2* and *Gsdm3* (*Gsdma 1*, *Gsdma 2*, Gsdma 3). Moreover, Gsdm3 played roles during the catagen to telogen transition at the end of hair follicle morphogenesis and the formation of hair follicle‐associated sebaceous glands.	^23^
	*DFNA5L* (*GSDMDC1, GSDMD*) gene was identified, together with *GSDM (GSDMA)*, *GSDML (GSDMB)*, *MLZE (GSDMC)*, *DFNA5* and their mammalian orthologs, all were found to constitute the *DFNA5 DC* (*GSDMDC*) family.	^24^
2006	*DFNB59* was found as the first human gene implicated in non‐syndromic deafness due to a neuronal defect.	^25^
2007	Pejvakin (PJVK) (encoded by *Dfnb59*) is essential for outer hair cell function.	^26^
2009	*GSDMB* was associated with asthma and autoimmune disease.	^27^
2010	GSDMD was first identified as a substrate of inflammatory caspase‐1 by enzymatic N‐terminal enrichment method with mass spectrometry‐based proteomics.	^28^
2012	*Gsdma3* mutation was associated with hair follicle keratinocytes and skin keratinocytes.	^29^
2012	Caspase‐11‐dependent macrophage death (pyroptotic cell) is detrimental to the host in the absence of caspase‐1 during *S. typhimurium* infection.	^30^
2014	Caspase‐4 and caspase‐5 act as direct sensors of cytosolic LPS.	^31^
2015	GSDMD was cleaved by inflammatory caspase1/4/5/11 and as the real executioner of pyroptosis.	^3^, ^32^, ^33^
2015	Pejvakin is essential for antioxidant activity of peroxisomes in hair cells and primary auditory neurons to protect the auditory system against noise‐induced oxidative stress.	^34^
2016	Liposome‐leakage and pore‐forming activities of the gasdermin‐N domain (GSDMD, GSDMA3 and GSDMA) are required for pyroptosis. The crystal structure of GSDMA3 was identified.	^35^
2017	GSDME was found as a substrate of caspase 3 to trigger pyroptosis under chemotherapy drugs treatment.	^36^
2018	Necrosulfonamide was identified as a direct chemical inhibitor of gasdermin D.	^37^
	GSDMD plays an essential function in the generation of neutrophil extracellular traps and NETosis.	^38^, ^39^
	ELANE could mediate GSDMD cleavage and induce lytic cell death in neutrophil.	^40^
	Cryo‐EM structure of the GSDMA3 membrane pore was found.	^41^
	Caspase‐8 was indicated to induce cleavage of GSDMD to activate pyroptosis during Yersinia infection.	^42^
2019	Caspase‐8 cleave GSDMD to promote lytic cell death during extrinsic apoptosis which could be counteracted by caspase‐3.	^43^
	Cathepsin G (CatG) could cleave GSDMD to induce pyroptosis in neutrophils and monocytes.	^44^
2020	GSDME‐triggered pyroptosis activated antitumor immunity. GZMB was found to directly cleave GSDME at the same site as caspase‐3 and then activate pyroptosis.	^6^
	GZMA could cleave GSDMB to induce pyroptosis in target cells.	^45^
	GSDMC could be specifically cleaved by caspase‐8 with macrophage‐derived TNFα treatment, which was switched by PD‐L1.	^46^
	*BRAFi* + *MEKi* treatment could promote cleavage of GSDME to regulate the tumor immune microenvironment.	^47^
	Succination blocked pyroptosis by inactivating GSDMD.	^48^
	FDA‐approved disulfiram identified as GSDMD inhibitors.	^49^
	Caspase‐6 was involved in pyroptosis in host defense against influenza A virus (IAV) infection.	^50^
	Substrate‐targeting mechanism was identified during recognition of GSDMD by inflammatory caspases.	^51^
2021	Cryo‐electron microscopy structures of the pore and the prepore of GSDMD was reported. GSDMD pore mediated preferential release of mature IL‐1 by electrostatic filtering.	^52^

## 2 THE MECHANISM OF THE PYROPTOSIS PATHWAY

**FIGURE 1:**
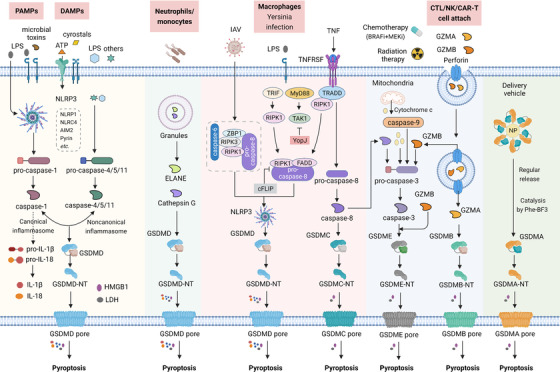
The molecular mechanism of pyroptosis activation. Under the stimulation of DAMPs and PAMPs, cytosolic canonical inflammasomes (NLRP3, NLRP1, NLRC4, AIM2, Pyrin, etc.) can respond to microbial infection (LPS, microbial toxins, etc.) or danger signals (ATP, crystals, etc.) to activate caspase‐1, while noncanonical inflammasomes directly respond to LPS or others to activate caspase‐4/5/11. After activation of inflammatory caspases, pro‐IL‐1β, pro‐IL‐18, as well as GSDMD is cleaved and liberates N‐terminal GSDMD (GSDMD‐NT) to form pores on the plasma membrane, companied with the release of inflammatory mediators (IL‐1β, IL‐18, HMGB1, LDH, etc.). ELANE in neutrophils and Cathepsin G in monocytes also directly cleave GSDMD to generate active GSDMD‐NT. Apoptotic caspase‐6 and caspase‐8 are also involved in NLRP3 inflammasome‐dependent GSDMD cleavage with IAV infection. *Yersinia* effector protein YopJ can inhibit TAK1 and cause caspase‐8‐dependent GSDMD cleavage. Besides, caspase‐8 also directly cleaves GSDMC to activate pyroptosis. Chemotherapy drugs, as well as cytochrome c released from mitochondria can induce caspase‐3‐dependent GSDME cleavage. Killing activity cells (CTL/NK/CAR‐T cells) can secret GZMA and GZMB, which are delivered by perforin into target cells and then cleave GSDME and GSDMB, respectively, to trigger pyroptotic cell death. GSDMA3 is used in the Phe‐BF3 desilylation bioorthogonal system to mediate delivery drugs into specific tumor cells Abbreviations: ATP, adenosine triphosphate; DAMP, damage‐associated molecular pattern; ELANE, neutrophil elastase; IAV, influenza A viruses; LDH, lactate dehydrogenase; LPS, lipopolysaccharide; PAMP, pathogen‐associated molecular pattern; TNF, tumor necrosis factor; TRADD, TNF receptor‐associated death domain; TRIF, Toll/IL‐1R domain‐containing adaptor‐inducing IFN‐beta.

Cell death is a complex and important regulatory network, which involves the immune system.^53^ The pyroptosis pathway is linked to both the innate immune system and the adaptive immune system, which contains varieties of molecules.^54^ Generally, gasdemin family members are core among the pyroptosis pathway, which can be cleaved and activated by inflammatory caspases (caspase‐1, caspase‐4, caspase‐5, caspase‐11), apoptosis‐related caspases (caspase‐3, caspase‐6, caspase‐8), and granzymes, especially granzyme A (GZMA) and granzyme B (GZMB).^3^, ^6^, ^36^, ^42^, ^45^, ^50^, ^55^, ^56^, ^57^, ^58^ Then, large amounts of cytokines and alarmins are released from the formed pores which exert effects on the downstream pathway.^7^, ^59^ Another important player is the inflammasome, although this is not the essential member in the pyroptosis pathway.^7^, ^59^, ^60^ Except for the above major components, there are also a lot of regulators working on each node of the pathway.^7^, ^49^, ^59^ (Figure 1)

### 2.1 The initiation of pyroptosis: Inflammasomes, caspases, and other enzymes

#### 2.1.1 Inflammasomes and caspase‐1

The inflammasomes are large multiprotein complexes which usually consist of pattern recognition receptors (PRRs), inflammatory caspases (caspase‐1) and sometimes an adaptor protein, like apoptosis‐associated speck‐like protein containing a CARD (ASC).^60^, ^61^, ^62^ Pyroptosis mediated by the inflammasomes is commonly categorized as the canonical caspase‐1‐dependent inflammasome pathway.^62^ Generally, as sensors to various dangers, the PRRs (NLRP1, NLRP3, NLRC4, NLRP6, NLRP7, NLRP9b, NLRP12, pyrin, and AIM2) first recognize a variety of stimulates, then activate pro‐caspase‐1 cleavage and ASC recruitment to assemble inflammasomes.^63^, ^64^, ^65^ Activated caspase‐1 can cleave pyroptosis executor gasdemin D (GSDMD) proteins (Asp275 site) to free the N‐terminal (NT) domain and generate nonselective pores on the cell membrane.^3^, ^55^, ^66^, ^67^ At the same time, caspase‐1 cleaves and activates the precursors of IL‐1β and IL‐18 to become mature IL‐1β and IL‐18. The latter together with other cellular contents then is released from the pores, leading to cell pyroptosis.^55^, ^60^, ^68^ In the case of NLRP3, the most common PRRs can be activated by a wide range of factors, including viruses, bacterial toxins, fungi, parasite, nucleic acids, crystalline substrates, some drugs, silica particles, long‐chain saturated fatty acids, reactive oxygen species (ROS) and various endogenous damage signals.^69^, ^70^, ^71^, ^72^ Notably, there is a two‐step procedure for NLRP3 inflammasome activation, including the priming step by various activators and the activating step through K^+^/Ca^2+^ efflux, mitochondria and lysosome‐related damages and etc.^73^ Also, NLRP1 responds to the *Bacillus anthracis* lethal toxin, *Toxoplasma gondii*, which activates assembly of the NLRP1 inflammasome and induces pyroptosis.^74^, ^75^, ^76^ Various NLR family apoptosis inhibitory protein (NAIP) proteins are essential for activation of the NLRC4 inflammasome, and human NAIPs directly link with flagellin and the type‐3 secretion system (T3SS) proteins. Comparably, NAIPs in mice can recognize the needle and inner rods of T3SS and flagellin.^77^, ^78^, ^79^, ^80^ Then, NAIPs recruit NLRC4 and induce its oligomerization to form the NLRC4 inflammasome.^81^, ^82^ Non‐NLRs, like AIM2, are activated by cytosolic double‐stranded DNA.^83^, ^84^, ^85^, ^86^, ^87^ Normally, pyrin is stimulated by the inactivation of small GTPase in the Rho family. For the Rho‐glucosylation activity of *Clostridium difficile (C. difficile)* toxin B (TcdB), *Clostridium botulinum* ADP‐ribosylating C3 toxin, and *Vibrio parahaemolyticus* VopS, some specific mutations like S208A and S242R, as well as the *Yersinia pestis* GTPase‐activating protein (YopE) and cysteine protease (YopT) can all activate the pyrin inflammasome.^88^, ^89^, ^90^, ^91^, ^92^ Other inflammasomes have been reviewed by others and not listed here.

#### 2.1.2 Caspase‐4/5/11

In contrast, the non‐canonical inflammasome pathway is independent of inflammasomes and directly activated by bacterial lipopolysaccharide (LPS) to cleave precursors of human caspase‐4, caspase‐5, and mouse caspase‐11.^56^, ^57^, ^58^, ^93^, ^94^ Then, the activated caspases can cleave GSDMs to rupture the plasma membrane, promote K^+^ efflux, and induce pyroptosis.^58^, ^95^ Sometimes, the procedure is also linked to activation of the NLRP3/caspase‐1 pathway, which ultimately promotes the maturation and release of IL‐1β and IL‐18, other than caspase‐4/5/11.^3^, ^57^, ^58^, ^96^ Besides, the pore formation does not always target the cytoplasmic membrane, and other membranes inside the cell, especially the mitochondrial membrane, are also involved.^97^ During inflammatory lung injury, the LPS‐mediated capspase‐11/GSDMD‐NT pathway can induce the formation of mitochondrial pores and the release of mitochondrial DNA (mtDNA) into endothelial cells (ECs). Then, the mtDNA activates the cyclic GMP‐AMP synthase/yes‐associated protein signaling pathway to damage endothelial regeneration.^98^

#### 2.1.3 Apoptotic caspases

In addition to inflammatory caspases (caspase‐1, caspase‐4, caspase‐5, caspase‐11), apoptosis‐related caspases (caspase‐3, caspase‐6, caspase‐8) are also involved in pyroptosis. Caspase‐3 can trigger gasdemin E (GSDME)‐dependent pyroptosis with tumor necrosis factor alpha (TNF‐α) treatment, chemotherapy drugs, and the high‐expression of GSDME.^6^, ^36^ The effector protein YopJ from *Yersinia* can activate the RIPK1/caspase‐8‐dependent GSDMD cleavage, resulting in pyroptotic cell death.^42^, ^99^, ^100^, ^101^ Under hypoxia, macrophage‐derived TNF‐α can induce pyroptosis through the caspase‐8/ gasdemin C (GSDMC) pathway, in a process which requires nuclear programmed death‐ligand 1 (PD‐L1). Furthermore, it is reported that antibiotic chemotherapy drugs also induce caspase‐8/GSDMC‐mediated pyroptosis in breast cancer.^46^ Generally, LPS usually induces caspase‐4/5/11‐related pyroptosis; however, a recent report has shown caspase‐8‐mediated pyroptosis in LPS‐activated macrophages. The process could be blocked by long isoform cFLIPL via promoting complex II formation.^102^ Recently, another apoptotic caspase, caspase‐6, was found to regulate the pyroptosis pathway by enhancing the interaction between receptor‐interacting serine/threonine protein kinase 3 and Z‐DNA‐binding protein 1, therefore, activating NLRP3/caspase‐1‐mediated pyroptotic signaling.^50^ Although there is no direct evidence that caspase‐9 can cleave GSDMs, it can cleave and activate caspase‐3, thus linking with GSDME indirectly.^103^ The roles of other caspases in pyroptosis pathway may be revealed by further and more in‐depth studies.^104^

#### 2.1.4 Other enzymes

Moreover, GSDMs are cleaved beyond different caspases. Granzyme family members including GZMA and GZMB are also proved to activate GSDM B (GSDMB) and GSDME, respectively.^6^, ^45^ Then, in neutrophils and monocytes, neutrophil elastase (ELANE) and Cathepsin G (CatG) cleave GSDMD to generate active GSDMD‐NT fragments and induce pyroptosis.^40^, ^44^

### 2.2 GSDM: The executors of pyroptosis

GSDMs have been identified as the executors of pyroptosis, which are protein families encoded by six paralogous genes in humans: GSDM A (*GSDMA*), GSDM B (*GSDMB*), *GSDMC*, *GSDMD*, *GSDME* (also known as *DFNA5*), and *DFNB59* (also known as *PJVK*). As for mice, they have three *Gsdma* genes (*Gsdma1*, *Gsdma2*, and *Gsdma3*), four *Gsdmc* genes (*Gsdmc1*, *Gsdmc2*, *Gsdmc3*, and *Gsdmc4*), one *Gsdmd* gene, and one *Gsdme* gene; however, they lack a *Gsdmb* gene. Their sequences share about 45% homology, with the exception of DFNB59. GSMDs A‐E all have two domains, an NT and a C‐terminal (CT) domain, which are capable of binding with each other by a flexible linker, while DFNB59 only possesses the NT domain.^105^ The CT domain restricts activation of the NT domain to maintain an auto‐inhibition status when the GSDM A‐E proteins keep the full‐length form. The NT domains will be activated after being separated from the CT domains; they polymerize and bind to acidic phospholipids in the inner membranes of the cells, form pores which serve as channels, releasing cytokines (e.g., IL‐1β, IL‐18), alarmins (e.g., adenosine triphosphate (ATP), and high‐mobility group box 1 (HMGB1)). Chemokines are released from pyroptotic cells into the extracellular space, damaging cell membrane integrity, leading to pyroptotic cell death and inducing inflammation in the tissues, after which innate or adaptive immune responses are activated to handle the emergencies (infection or damage).^35^ A pore formation model of the GSDM family was constructed using the crystal structure analysis for full‐length of mouse GSDMA3 and cryo‐electron microscopy structure analysis of the GSDMA3‐NT pore,^41^ as the function of GSDM proteins generally depends on their structural conformation.^106^

**FIGURE 2:**
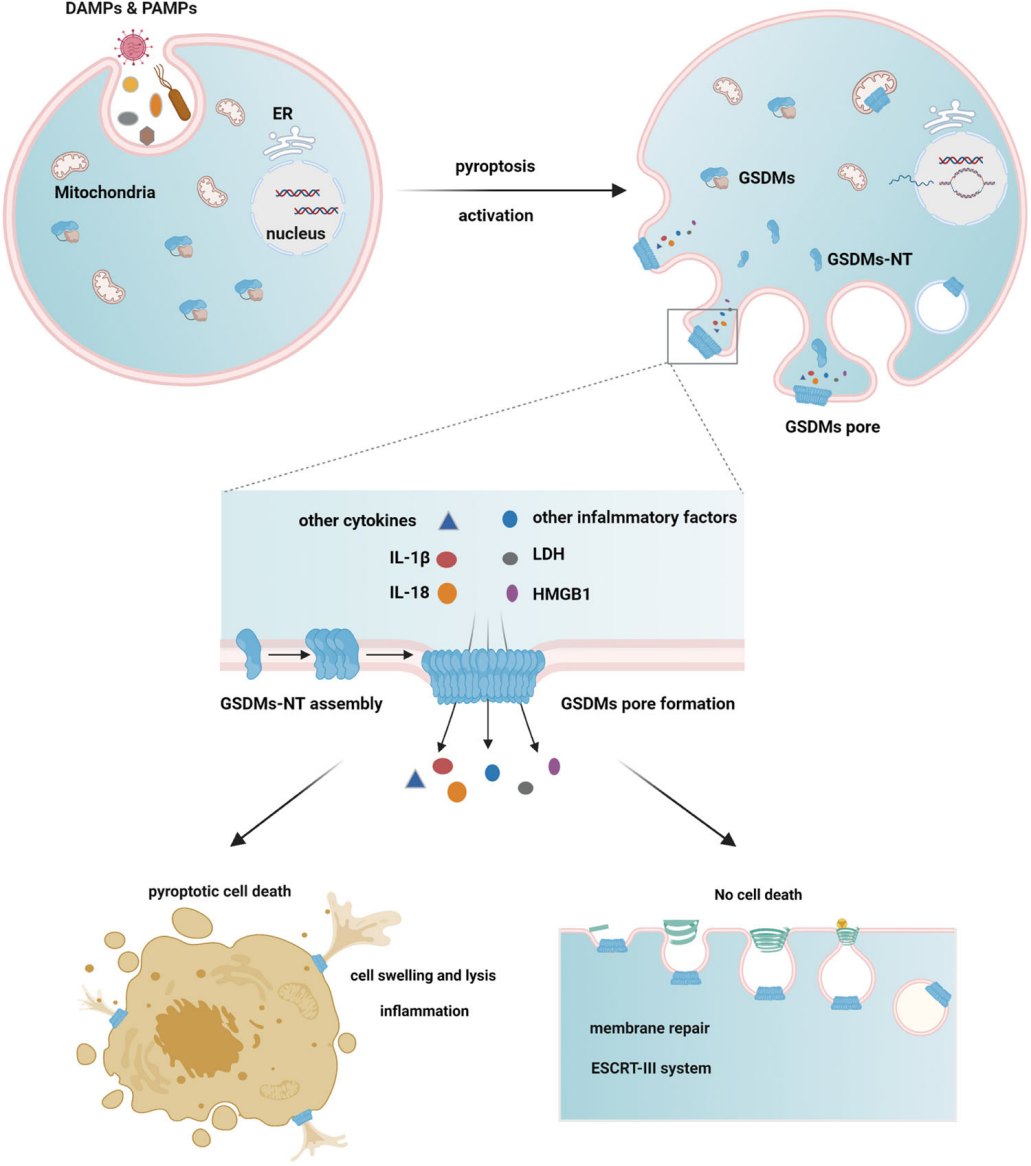
GSDMs function as executors of pyroptosis and link with or without cell death. In response to various infective pathogens and danger signals, GSDMs are activated and cleaved by different enzymes, liberating NT domains to assembly GSDMs complex on the plasma membrane and cause pore formation. Through which inflammatory factors and other cellular contents are released. Then pyroptotic cell death occurs with membrane bubbles and cell flattening. However, some cells will initiate ESCRT‐III‐dependent membrane repair to fight against the membrane damage, thus avoiding cell death Abbreviation: ESCRT, endosomal sorting complexes required for transport.

Lytic cell death does not always occur after pore formation caused by GSDMs, and cell membrane repair can also be initiated in some surviving cells by recruiting the ESCRT complex to the damaged membrane region. The ESCRT‐III system negatively regulates the pyroptosis signaling pathway and may provide new insights into the general cellular survival and self‐repair mechanisms during ICD.^4^, ^107^ (Figure 2) (Table 2).

**TABLE 2:** The features of GSDMs genes

Human gasdermin (chromosomal location)	Mouse gasdermin (chromosomal location)	Domains	Activating proteolytic cleavage	Pore‐forming activity	Membrane targeting	Tissue expression	Biological functions	Related cancers	Other related diseases	Refs.
*GSDMA* (chr 17q21.1)	*Gsdma1* *Gsdma2* *Gsdma3* (chr 11D)	N‐GSDM C‐GSDM	?	Yes	Plasma membrane	Esophagus, gastric, skin	?	Gastric cancer	Systemic sclerosis in humans, alopecia in mice	^19^, ^108^, ^109^, ^110^, ^111^
*GSDMB* (chr 17q21.1)	–	N‐GSDM C‐GSDM	Caspase‐1 GZMA	Yes	?	Airway, esophagus, gastrointestinal tract, liver and colon epithelium, neuroendocrine cells, immune cells (T cells)	?	Colon, rectal, pancreatic, cervical, breast, lung, liver cancers	Inflammatory bowel disease, asthma, type I Diabetes, Systemic sclerosis	^45^, ^112^, ^113^, ^114^, ^115^, ^116^, ^117^, ^118^, ^119^, ^120^, ^121^
*GSDMC* (chr 8q24.21)	*Gsdmc1* *Gsdmc2* *Gsdmc3* *Gsdmc4* (chr 15D1)	N‐GSDM C‐GSDM	Caspase‐8	Yes	?	Keratinocytes, trachea, spleen, esophagus, gastric, small intestine, caecum, and colon	?	Colorectal cancer and melanoma, lumbar spinal stenosis	?	^46^, ^122^, ^123^, ^124^, ^125^
*GSDMD* (chr 8q24.3)	*Gsdmd* (chr 15D3‐E1)	N‐GSDM C‐GSDM	Caspase‐1/4/5/11 Caspase‐8 Cathepsin G ELANE	Yes	Plasma membrane, Nucleus, Mitochondria, Neutrophil granules, LC3+ autophagosomes	Immune cells, placenta, esophagus and gastrointestinal tract epithelium	Pyroptosis NETosis	Esophagus, gastric, pancreatic, prostate cancers, melanoma, salivary gland tumors, Jurkat T cells, Ramos B cells	Sepsis, experimental autoimmune encephalomyelitis, macular degeneration, neonatal onset multisystem inflammatory disease in mice, liver fibrosis, inflammatory bowel disease	^35^, ^40^, ^44^, ^60^, ^99^, ^126^, ^127^, ^128^, ^129^, ^130^, ^131^, ^132^, ^133^
*GSDME/DFNA5* (chr 7p15.3)	*Gsdme/Dfna5* (chr 6B2.3)	N‐GSDM C‐GSDM	Caspase‐3 GZMB	Yes	Plasma membrane, Mitochondria	Cochlea, placenta, heart, brain, kidney	Pyroptosis	Gastric, colorectal, breast cancers (Inactivated by DNA methylation), melanoma	Autosomal dominant nonsyndromic hearing loss, acute kidney injury, obstructive nephropathy, drug‐induced nephrotoxicity	^18^, ^36^, ^47^, ^134^, ^135^, ^136^, ^137^, ^138^, ^139^
*DFNB59* */PJVK* (chr 2q31.2)	*Dfnb59/Pjvk* (chr 2C3)	N‐GSDM Zinc finger	?	?	Peroxisomes	Brain, eye, inner ear, heart, lung, kidney, liver, intestine, testis	Hair cell maintenance	?	Recessive nonsyndromic hearing impairment, autosomal recessive auditory neuropathy	^25^, ^26^, ^34^, ^140^, ^141^

*Note*: −, absent; ?, unknow; C‐GSDM, C‐terminal of gasdermin; N‐GSDM, N‐terminal of gasdermin.

#### 2.2.1 GSDMA

GSDMA is the first GSDM family member, which was originally named GSDM1; its gene is located on human chromosome 17q21.1, while the *Gsdma1*, *Gsdma2*, and *Gsdma3* genes are clustered on mouse chromosome 11D. GSDMA is primarily detected in the upper gastrointestinal tract, epithelium of the skin, and the lung,^126^, ^142^ while it is generally silenced in gastric cancer cells.^19^ GSDMA is upregulated by transforming growth factor‐beta to induce apoptosis in pit cells of the gastric epithelium.^111^

The molecular mechanism of GSDMA is not well known until now. It may play roles in stress responses due to its expression in the suprabasal layer, differentiating cells and the epidermis outside the tumor.^143^ Asthma‐associated polymorphisms at the locus 17q12‐21 mediate the expression and methylation of the *GSDMA* gene in CD4^+^ T cells.^108^ In turn, the *GSDMA* polymorphism (rs3859192 on chr17q21) is strongly linked with asthma susceptibility and intermediate phenotypes in asthma.^142^, ^144^ Also, the *GSDMA* variant (rs3894194) also modulates systemic sclerosis (SSc) susceptibility in monocyte‐derived macrophages (MDMs).^109^

Among the three *Gsdma* genes in mice, mutations of *Gsdma3* are hotspots, which not only cause alopecia during the formation of hair follicle‐associated sebaceous glands, as well as the transition from catagen to telogen at the final period of hair follicle morphogenesis,^23^ but display obvious phenotypes in epidermal hyperplasia and progressive hair loss.^145^ Gain‐of‐function mutations in the CT domains of GSDMA3 reveal the function of its NT fragments. The GSDMA3 Y344H mutant protein and NT domain induce autophagy with mitochondria and ROS generation.^146^, ^147^ GSDMA3 regulates mitochondrial homeostasis by impinging on the mitochondria via heat shock protein 90 (HSP90), inducing oxidative stress and mitochondrial permeability transition.^110^

#### 2.2.2 GSDMB

GSDMB is found on human chromosome 17q21.1, while no *Gsdmb* gene has been identified in mice. Human GSDMB has six isoforms (Q8TAX9, UniProt). There are differences in the length and sequence of the linkers between the CT and NT domains of isoforms 1–4 and isoform 6, whereas there is no NT domain in isoform 5.^106^ GSDMB is increasingly expressed in multiple tissues, especially the upper gastrointestinal epithelium,^148^ liver and colon epithelium,^148^ airway epithelial cells,^149^ neuroendocrine cells,^148^ and immune cells (T cells).^45^, ^115^, ^150^

Highly expressed GSDMB is also linked to tumor aggressiveness in several types of cancers, such as gastric, hepatic, colon, and cervical cancer,^124^, ^148^, ^151^ as well human epidermal growth factor receptor 2 (HER2)‐positive breast cancer with poor prognosis,^106^, ^118^, ^152^, ^153^ and pancreatic cancer susceptibility (false discovery rate ≤ 0.05),^117^ implying the role of GSDMB as an oncogene and potential attractive therapeutic target in metastasis, poor prognosis, and drug resistance to anti‐HER2 therapy.

Interferon‐gamma (IFN‐γ) upregulates the expression of GSDMB, lymphocyte‐derived GZMA cleave GSDMB between the CT and NT linkers at the Lys^244^ site, leading to pyroptosis in GSDMB‐positive cells.^45^ Caspase‐1 cleaves GSDMB at the aspartate 236 (D236) position, inducing pyroptosis in airway epithelial cells.^149^ Like GSDMD, GSDMB also could be cleaved by apoptotic caspase‐3, 6, 7 within the NT domain, therefore, losing pyroptotic ability,^106^ indicating the dependence of GSDM function on specific structural forms.

Genome‐wide sequencing and functional genes indicate that single nucleotide polymorphisms of GSDMB are related to the increased susceptibility of ulcerative colitis and Crohn's disease, as well as asthma exacerbations and antiviral pathways.^106^, ^112^, ^149^ Analogous to GSDMA, GSDMB is also linked to childhood‐onset asthma,^154^ while *GSDMB* (rs9303277) in CD4^+^ T cells is an SSc‐associated susceptibility locus.^115^ In addition, GSDMB is included in the Type 1 and 2 diabetes (T1D, T2D) islet expression quantitative trait loci interaction network.^114^

#### 2.2.3 GSDMC

GSDMC is originally isolated from melanoma cells, so it is known as a melanoma‐derived leucine zipper extranuclear factor (MLZE), whose gene is located on human chromosome 8q24.21. The homolog of *GSDMC* in mice is composed of four members, *Gsdmc 1*, *Gsdmc 2*, *Gsdmc 3*, and *Gsdmc 4*, which are clustered on mouse chromosome 15 (15D1).^126^ The GSDMC is preferentially found in the epithelium of the gastrointestinal tract and skin,^126^ spleen,^20^ and metastatic melanoma cells,^20^ as well as lung and colorectal cancer (CRC) tissues.^125^, ^155^

Ultraviolet irradiation can induce the expression of GSDMC mediated by the calcium/calcineurin/nuclear factor of activated T‐cells, the cytoplasmic 1 (NFATc1) pathway and the extracellular signal‐regulated kinase (ERK)/c‐Jun N‐terminal kinase (JNK)/matrix metalloproteinase 1 pathway in human skin keratinocytes.^156^, ^157^ Genome‐wide association studies show that GSDMC is associated with chronic back pain,^158^ *Mycoplasma hyopneumoniae (M. hyo)* Ab variations in pigs,^159^ lumbar spinal stenosis,^122^ multi‐type methylation in subchondral bone cartilage and osteoarthritis‐related cartilage,^160^, ^161^ sciatica derived from lumbar disc herniation,^162^ and monocyte counts.^163^

GSDMC functions as an oncogene, mediating varieties of progressive processes involving types of tumors. GSDMC may serve as a significant therapeutic target in CRC patients as it promotes tumor cell proliferation in CRC by reducing activation of the transforming growth factor β receptor type II.^125^ GSDMC is hypomethylated in lung adenocarcinoma (LUAD) cells, and its upregulation implies poor prognosis in LUAD patients.^155^ Also, GSDMC is linked to metastasis in malignant melanoma,^123^, ^164^ and the high expression of GSDMC is related to the poor survival of breast cancer. Upon hypoxia, the GSDMC gene is transcriptionally activated for nPD‐L1/p‐Y705‐signal transducer and activator of transcription 3 (STAT3) complex binding to the STAT3‐binding site in the promoter region of *GSDMC*. Experiments *in vivo* have shown that GSDMC, along with nuclear PD‐L1 and caspase‐8, is required in the tumor necrosis induced by TNF‐α which are derived from macrophages.^46^ *GSDMC* variants at the chromosome 8q24 locus also modulate genetic risk to several cancers, including prostate cancer.^165^, ^166^ However, GSDMC, whose expression is suppressed, can inhibit the proliferation of gastric and esophageal cancer cells. It is suggested that GSDMC has the potential to serve as a tumor suppressor.^124^ Therefore, the roles of GSDMC might serve as an organ‐specific feature, and its function in carcinogenesis is not very clear, virtually.

#### 2.2.4 GSDMD

*GSDMD* is located on human chromosome 8q24.3, while *Gsdmd* is found on mouse chromosome 15D3‐E1. GSDMD is first identified as the efficient substrate of inflammatory caspases among the GSDM family members.^28^ In 2015, GSDMD was shown to be the real executor of inflammasome‐induced pyroptosis.^3^, ^33^ Since the key turning point, the functional role and molecular features have been gradually revealed. GSDMD is expressed in various cells, including the gastrointestinal epithelium, placenta, and immune cells, especially macrophages and dendritic cells (DCs), as well as cancers, such as esophageal and gastric cancer, melanoma, pancreatic cancer, prostate cancer, salivary gland tumor, and Jurkat T, and Ramos B cancer cell lines.^24^, ^124^, ^167^, ^168^

Although GSDMD is the first effector protein to be found in pyroptosis and has been studied the most frequently, the perspicuous regulatory mechanism is not well known. IFN regulatory transcription factor‐2 (IRF2) is found to transcriptionally regulate GSDMD expression.^169^ Given that IRF2 is reported as a transcription factor which regulates IFN and IFN‐inducible genes, and the down‐regulation of IRF2 in CRC cells is associated with immune suppression and immunotherapy resistance,^170^ the IRF2‐GSDMD regulatory axis may link GSDMD to immune activity in the tumor microenvironment (TME).

GSDMD is found to be cleaved by inflammasome‐activated caspase‐1 and LPS‐activated caspase‐4/5/11. However, the way in which GSDMD is recognized specifically by these caspases is not clear. Until recently, crystal structural analysis of caspase‐1‐GSDMD‐C complex and caspase‐4/11‐GSDMD‐C complex has revealed the specific recognition mode.^51^ In contrast to other GSDM members, there have been no specific disease‐related mutants of GSDMD reported, which implies that GSDMD plays a broad‐spectrum role in hosts.

The pyroptosis pathway mediated by GSDMD can also be limited in different ways. For example, disulfiram is reported to specifically inhibit pore formation by modification at the Cys191/Cys192 sites of GSDMD in human/mouse, but this does not disturb the processing of IL‐1β and GSDMD, thereby blocking IL‐1β release and GSDMD‐mediated pyroptosis.^49^ In contrast, mafenide is reported to prevent the cleavage of GSDMD by binding to the Asp275 site of GSDMD directly, reducing NT fragments and weakening pyroptosis.^171^ Mouse models have showed that GSDMD exerts the effect in many inflammatory disorders, such as endotoxemia and sepsis, experimental autoimmune encephalomyelitis, Familial Mediterranean Fever, and macular degeneration and neonatal onset multisystem inflammatory disease.^32^, ^132^, ^133^, ^172^, ^173^

#### 2.2.5 GSDME/DFNA5

*GSDME*, also known as *DFNA5*, is located on human chromosome 7p15.3, while *Gsdme*, also known as *Dfna5*, is found on mouse chromosome 6B2.3. *GSDME* mutation was first identified to cause an autosomal dominant hearing impairment.^18^, ^174^, ^175^ GSDME is mostly expressed in the cochlea, placenta, brain, female reproductive tract (especially the placenta), kidney, heart, and muscle.^36^ In contrast to non‐tumoral tissues, expression of GSDME is limited in most cancers including gastric cancer, melanoma, CRC, invasive breast cancer, and other human cancers.^36^, ^138^ GSDME suppression in cancer cells is most dependent on the DNA methylation of the GSDME promoter.^138^, ^164^, ^175^ The inactivation of GSDME conforms to the clinical and animal results that lower the expression of GSDME and is associated with a poor 5‐year survival and enhanced metastases in breast cancer.^6^, ^164^, ^175^ All of this suggests that GSDME might be a tumor suppressor and worth in‐depth exploration. However, there are also various loss‐of‐function (LOF) mutations of GSDMD in different cancers, although this is less common than the epigenetic suppression of GSDME. Thus, LOF mutations together with the epigenetic suppression of GSDME might be two arch strategies developed by cancer cells to escape GSDME‐mediated tumor suppression.^36^, ^164^, ^175^ The transcription of GSDME may be regulated by p53, which is also inactivated in many cancer cells, as virtually nothing is known about the exact regulatory details.^176^ Generally, caspase‐3 is reported to specifically cleave GSDME, leading to GSDME‐mediated pyroptotic cell death when viral infection or chemotherapy drugs are used. Given that GSDME is also expressed in normal cells and tissues, chemotherapy drug‐induced pyroptosis might also cause damage to the host,^36^, ^177^, ^178^ while 2‐bromopalmitate might inhibit chemotherapy‐induced pyroptosis.^178^

#### 2.2.6 DFNB59/PJVK

*DFNB59*, also known as *Pejvakin* (*PJVK*), is located on human chromosome 2q31.2 and comprises seven exons,^179^ while *Dfnb59*, also known as *Pjvk* is found on mouse chromosome 2C3. PJVK encoded by *DFNB59*, is a protein (352 residues) which is distantly related to other GSDM protein family members.^19^, ^179^ PJVK expression has been found in outer hair cells, as well as supporting cells, hair cells, and cell bodies of spiral ganglion neurons (SGNs) in the inner ear.^25^, ^26^ PJVK is also critical for protection of the architecture of stereocilia and the function of hair cells and SGNs.^34^, ^140^, ^180^, ^181^, ^182^, ^183^

*DFNB59*, the same as its paralog *DFNA5*, is also reported to be associated with progressive hearing loss. *DFNB59* is first identified as the causal gene of autosomal recessive deafness, which also plays key role during the signal transmit of auditory nerve.^25^ As a hearing‐loss gene, variants of *DFNB59* are linked with non‐syndromic hearing loss.^141^, ^184^, ^185^, ^186^, ^187^ In addition, case reports have reported that PJVK also plays a role in other hearing‐related syndromes like auditory neuropathic spectrum disorder (ANSD) and poor cochlear implants performance.^25^, ^188^

## 3 THE RELATIONSHIP BETWEEN PYROPTOSIS AND METABOLISM

Pyroptosis is accompanied by inflammasome activation, GSDM and caspase cleavage, and cytokine releases, leading to inflammatory death in cells and expending inflammation in multi‐tissues; all of these processes have impacts on a variety of metabolic disorders in different organs, including the liver, cardiovascular system, allergic and autoimmune diseases, diabetes, obesity, and others. In the case of the most studied NLRP3 inflammasome, which is a critical sensor of nutrient overload, this can be activated by various metabolic danger signals, such as uric acid crystals in gout,^189^, ^190^, ^191^ cholesterol crystals and oxidized low‐density lipoprotein (ox‐LDL) in atherogenesis,^192^, ^193^, ^194^, ^195^ and glucose, fatty acids and islet amyloid polypeptide in T2D.^196^, ^197^, ^198^, ^199^

**FIGURE 3:**
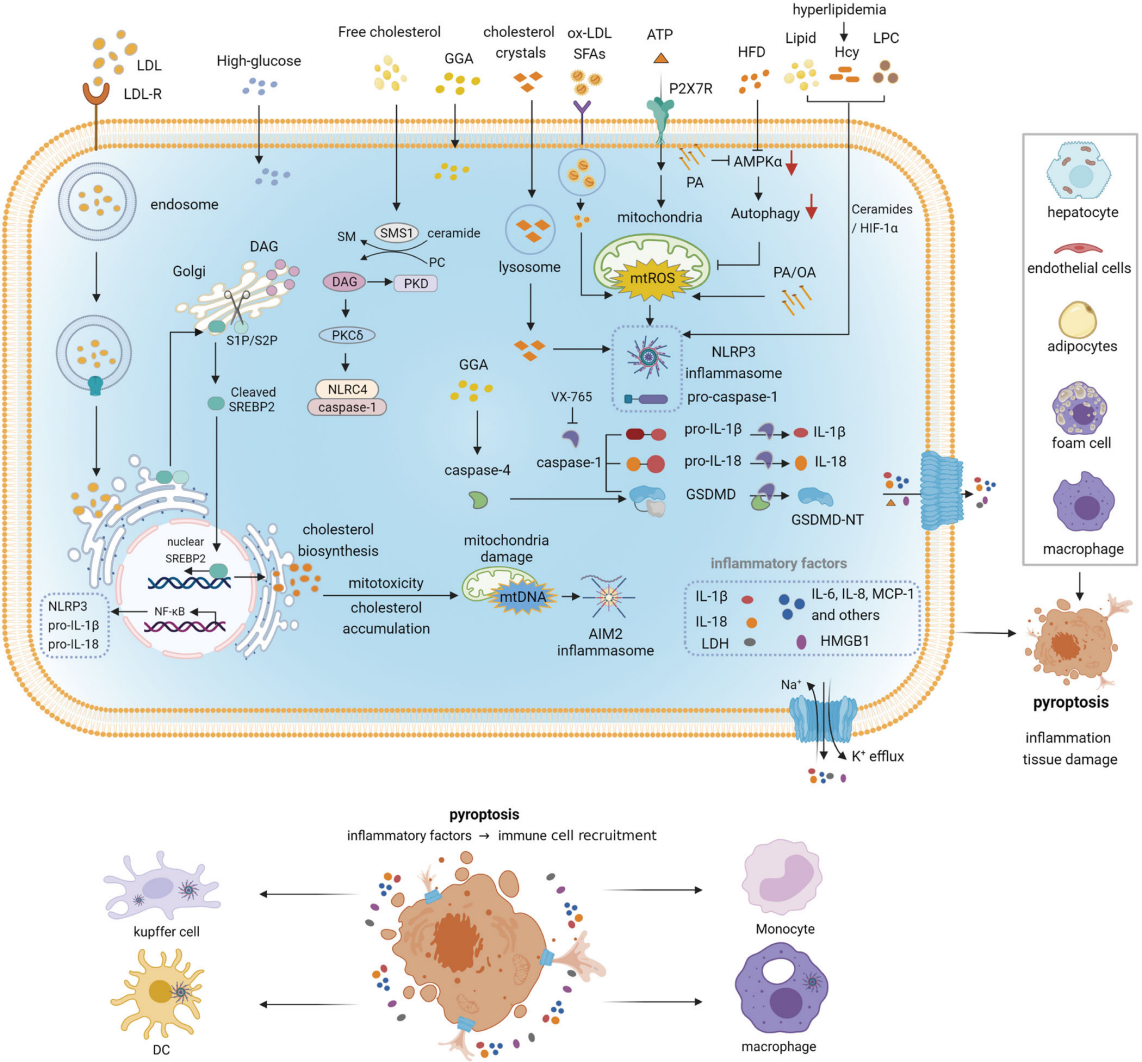
Pyroptosis induced by different metabolic signals. Various metabolic signals such as cholesterol crystals, ox‐LDL, ATP, lipid, Hcy, LPC, PA, and OA can trigger NLRP3 inflammasome‐dependent GSDMD cleavage and induce pyroptosis. Massive inflammatory mediators released by pyroptotic cells cause inflammation in different organs companied with tissue damages in various degrees. Then immune cells are recruited and infiltrated in the inflammatory regions. Abbreviations: DAG, diacylglycerol; GGA, geranylgeranoic acid; Hcy, homocysteine; LPC, lysophosphatidylcholine; OA, oleic acid; ox‐LDL, oxidized low‐density lipoprotein; PA, palmitic acid; SM, sphingomyelin; SMS1, sphingomyelin synthases; SREBP2, sterol regulatory element‐binding protein 2

In response to infective pathogens and dangerous signaling molecules, profound metabolic fluctuation normally appears in immune cells.^200^, ^201^, ^202^, ^203^ Beyond supplying energy, various nutrients can generally activate immune cells by serving as signaling molecules. For instance, fluctuating concentrations of glucose as an energy supplement stimulate insulin secretion from islet cells and the release of IL‐1β from macrophages.^204^ In turn, both insulin and IL‐1β can regulate glucose concentrations by stimulating glucose uptake in fat and muscle. Meanwhile, they activate the immune system via accelerating the absorption of glucose into immune cells. In addition, the deletion of macrophages could damage the IL‐1β‐mediated consumption of glucose in T‐cell‐ and B‐cell‐deficient *Rag2^−/−^* mice.^205^ Besides, there is a protein kinase B (AKT)‐dependent glycolytic potential for CD8^+^ memory T cells, which is responsible for rapid IFN‐γ recall responses^200^ (Figure 3).

### 3.1 Liver diseases

Sulfatide is a type of 3‐O‐sulfogalactosylceramide, whose production is dependent on cerebroside sulfotransferase and ceramide galactosyltransferase. As this has a multifunctional role in diverse biological fields, abnormal expression or metabolic changes to sulfatide correlate with many different types of diseases including cancer.^206^ Abnormal lipid accumulation in hepatocytes is a feature of non‐alcoholic fatty liver disease (NAFLD). Long‐term high‐fat diet (HFD) and palmitic acid (PA) significantly upregulate the expression of pyroptosis‐related molecules including NLRP3, GSDMD, GSDMD‐NT, caspase‐1, cleaved caspase‐1 (p20), IL‐1β, and IL‐18 in NAFLD.^207^ In addition, as one of the free fatty acids, PA also has a strong stimulatory effect on apoptosis,^207^ while oleic acid (OA) absorbs the accumulation of triglycerides, inducing abundant lipid accumulation.^208^ Both OA and PA can activate caspase‐1‐mediated pyroptosis in steatotic hepatocytes. The process could also be accompanied by P2 × 7 receptor (P2 × 7R) activation.^209^ A possible explanation is that PA‐related pyroptosis may contribute to endoplasmic reticulum (ER) stress in HepG2 cells, and OA is capable of improving hepatocellular lipotoxicity both *in vitro* and *in vivo*.^210^ Sphingomyelin synthases (SMSs) could generate sphingomyelin (SM) and diacylglycerol (DAG), while free cholesterol and SMSs in hepatocytes are found to mediate hepatocyte pyroptotic injury through DAG‐PKCδ‐NLRC4 axis.^211^ It has been reported that geranylgeranoic acid (GGA) (C20:4) could induce pyroptotic cell death in human hepatoma‐derived HuH‐7 via toll‐like receptor 4 (TLR4) signaling pathway. In fact, GGA is a branched‐chain polyunsaturated fatty acid, an endogenous metabolite derived from the mevalonate pathway in mammals.^212^

### 3.2 Cardiovascular diseases

Pyroptosis is activated by various pathological conditions, which are known risk factors of atherosclerosis, including oxidative stress, hyperglycemia, dyslipidemia, and inflammation.^192^, ^193^, ^213^ It is the first time that pyroptosis in vascular ECs has been reported to correlate with atherosclerotic progression, which can be partly attributed to ox‐LDL.^214^ It has been found that ox‐LDL could induce pyroptosis via the ROS‐dependent pathway in ECs. Then, fibroblast growth factor 21 is reported to inhibit ox‐LDL‐induced pyroptosis and related molecules in human umbilical vein ECs (HUVECs) through the Tet methylcytosine dioxygenase (TET2)/ubiquinol cytochrome c reductase core protein I (UQCRC1)/ROS pathway.^194^, ^215^ Ox‐LDL also induces pyroptosis in vascular smooth muscle cells (VSMCs) through activation of the NLRP3 inflammasome. VX‐765 can significantly reduce ox‐LDL‐induced pyroptosis and IL‐1β processing in VSMCs.^216^ Like ox‐LDL, the saturated fatty acid PA also triggers pyroptosis in an ROS‐dependent manner in HUVECs, and the PA‐induced pyroptosis can be limited by dihydromyricetin, which is inseparable from activation of the nuclear factor E2‐related factor 2 (NRF2)‐antioxidant response element signaling pathway.^217^

It is known that pyroptotic cell numbers decrease following pretreatment with colchicine and cholesterol crystals in HUVECs, and the limitation to cholesterol crystals by colchicine can function through AMP‐dependent kinase (AMPK)/ Sirtuin1 (SIRT1) signaling.^193^ Caspase‐1 activation by hyperlipidemia promotes EC activation and monocyte recruitment into the arterial intima, resulting in the upregulation of NLRP3, caspase‐1, and IL‐1β, and leads to pyroptosis in ECs.^214^, ^218^ It is suggested that pyroptosis induced by the NLRP3 inflammasome also participates in the accumulation of atherosclerotic plaques.^219^ Thus, in mice, hyperlipidemia aggravates ECs accompanied with aortic atherosclerosis.^220^, ^221^, ^222^

Apolipoprotein‐M and sphingosine‐1‐phosphate (S1P) can restrain TNF‐α‐induced pyroptosis by binding to S1P receptor 2 (S1PR2), thereby attenuating atherosclerosis.^223^ As for estrogen, it inhibits pyroptosis in vascular ECs mainly through the estrogen receptor alpha (ER‐α)‐mediated activation of autophagy. The reaction can ameliorate atherosclerosis as early as possible, usually before the menopausal stage.^224^ Trimethylamine N‐oxide (TMAO) is usually generated from the phosphatidylcholine metabolism of gut flora, which also presents as a danger signal for cardiovascular diseases. TMAO promotes ROS‐induced pyroptosis in vascular ECs through the upregulation of the succinate dehydrogenase complex subunit B (SDHB), thereby contributing to the progression of atherosclerotic lesions.^225^ Homocysteine (Hcy) as an independent risk factor of atherosclerosis can facilitate IL‐1β and IL‐18 secretion, breaking the endothelial barrier at early stages. The amount of Hcy, NLRP3, cleaved caspase‐1, GSDMD, IL‐1β, and IL‐18 in the vascular endothelium of the ascending aorta are significantly upregulated in postmenopausal females in comparison with pre‐menopausal ones.^226^, ^227^ Hcy, together with LPS, could activate pyroptosis in ECs by stimulating the caspase‐1‐dependent inflammasome.^228^ Lysophosphatidylcholine (LPC), one of the key lipid components composed of ox‐LDL and the cell membrane, plays a key role in atherosclerosis with an extensively pro‐inflammatory activity. LPC promotes the formation of foam cells, among others, which is dependent on the NLRP3 inflammasome to increase the biogenesis of lipid droplets. Furthermore, pyroptosis, inflammasome activation, and IL‐1β secretion induced by LPC in both cells are largely inseparable from potassium efflux as well as lysosomal damage in human monocytes.^229^

### 3.3 Diabetes

Hyperglycemia is the obvious feature of diabetes, which is a complex metabolic syndrome. Hyperglycemia induces pyroptosis in diabetic cardiomyopathy by upregulating miR‐30d.^230^ Beyond inflammation, the NLRP3 inflammasome is also linked to the regulation of glucose homeostasis.^231^ Sodium butyrate (NaB) could ameliorate damage to renal glomerular ECs induced by high‐glucose (HG) through the caspase1/GSDMD pathway.^199^ Besides, it is NaB, rather than LPS, which triggers gingival epithelial cell pyroptosis, a form of pro‐ICD, and caspase‐3/GSDME contributes to this effect. Pyroptotic cell death upregulates IL‐8 and monocyte chemotactic protein‐1, provoking inflammatory responses by releasing intracellular contents into the extracellular microenvironment.^232^

HG insult causes damage to adipose function. Metformin and resveratrol could suppress dynamin‐related protein 1 activity and prevent activation of the NLRP3 inflammasome by limiting ER stress to reverse the damage process and protect mitochondrial integrity.^233^ The cause of hyperglycemia associated with diabetic nephropathy (DN) is insufficient insulin secretion or insulin resistance, resulting in hypoxia and the excessive production of inflammatory cytokines. HG conditions in diabetic kidney disease could induce the increased expression of TLR4, cleaved caspase‐1, GSDMD‐NT and secretion of IL‐1β, and IL‐18; pyroptosis in these HG conditions could be partly reversed by TLR4 inhibitors (TAK‐242) and NF‐κB inhibitors (parthenolide).^234^ Inflammation, oxidative stress and pyroptosis induced by HG in HK‐2 cells could be inhibited by the long noncoding RNA KCNQ1OT1/miR‐506‐3p pathway, regulating the progression of DN.^235^ Moreover, in HG‐induced renal tubular cells, inflammation, oxidative stress and pyroptosis are limited by the overexpression of long noncoding RNA GAS5 and the down‐regulation of miR‐452‐5p.^236^ In HG‐stimulated podocytes, the release of pro‐inflammatory cytokines and chemokines, including intracellular ROS, IL‐6, and IL‐1β, is increased through the activation of TLR4/NF‐κB signaling.^237^, ^238^

### 3.4 Obesity

Obesity is strongly associated with low‐grade inflammation in whole bodies and adipose tissue, with adipocyte death in both mice and human subjects reported to be at a 30‐fold increased level. This cell death is partly due to the pyroptosis of adipocytes caused by macrophages.^239^ Obesity itself also facilitates assembly of the NLRP3 inflammasome in adipose tissue macrophages, which could induce macrophage‐mediated T cell activation and IFN‐γ release.^240^ Adiposity and insulin sensitivity could be regulated by the NLRP3 inflammasome during obesity,^196^ demonstrating improved glucose homeostasis in mice lacking *Nlrp3* under HFD.^241^ Hypertrophic adipocytes likely induce obese adipocyte death by pyroptosis mediated by NLRP3‐dependent caspase‐1 activation in leptin‐deficient *ob/ob* mice and obese humans, as active caspase‐1 is detected in the cytoplasm of some hypertrophic adipocytes. Also, adipose tissue inflammation is linked to the death of hypertrophic adipocytes.^242^, ^243^ Obesity is normally linked to the metabolic infiltration of MDMs in multiple tissues, and monocytes from obese individuals usually present with the elevated activity of inflammatory caspases. The saturated fatty acid palmitate, but not palmitoleate, can activate the pyroptotic death of monocytes through caspase‐4/5, causing inflammatory mediators release and inflammasome activation.^244^

### 3.5 Others

Non‐esterified fatty acids are reported to induce pyroptosis and inflammation via the NLRP3 inflammasome and TLR4/NF‐κB pathway, which can be alleviated by N‐acetylcysteine.^245^ Docosahexaenoic acid (DHA), a type of Omega‐3 fatty acid, can modulate inflammation and present anticancer effects. DHA has been reported to activate the caspase‐1/GSDMD/IL‐1β pathway to induce pyroptosis in triple‐negative breast cancer.^246^ Recently, it has been found that dimethyl fumarate or endogenous fumarate could modify GSDMD and GSDME at critical residues, which will prevent the interaction between GSDMD and caspases, limit its processing, oligomerization, and the ability of pore formation and inducing pyroptotic cell death. The finding provides the possibility of fumarate‐based therapeutics in the treatment of multiple sclerosis.^48^ The excessive deposition of uric acid crystals is usually associated with gout and accompanied by the obvious activation of NLRP3 inflammasomes in the inflammatory disease.^189^, ^190^ Uric acid crystals (monosodium urate)‐induced pyroptosis functions in the acute gouty arthritis, possibly through regulating the NF‐κB/NLRP3/GSDMD signaling pathway.^191^ Prostaglandin E2 (PGE2) can inhibit caspase‐11‐dependent pyroptosis in murine and human macrophages, implicating PGE2 as a negative regulator of caspase‐4/11‐driven pyroptosis to contribute to allergic airway inflammation.^247^

## 4 PYROPTOSIS AND TUMOR IMMUNE MICROENVIRONMENT

**FIGURE 4:**
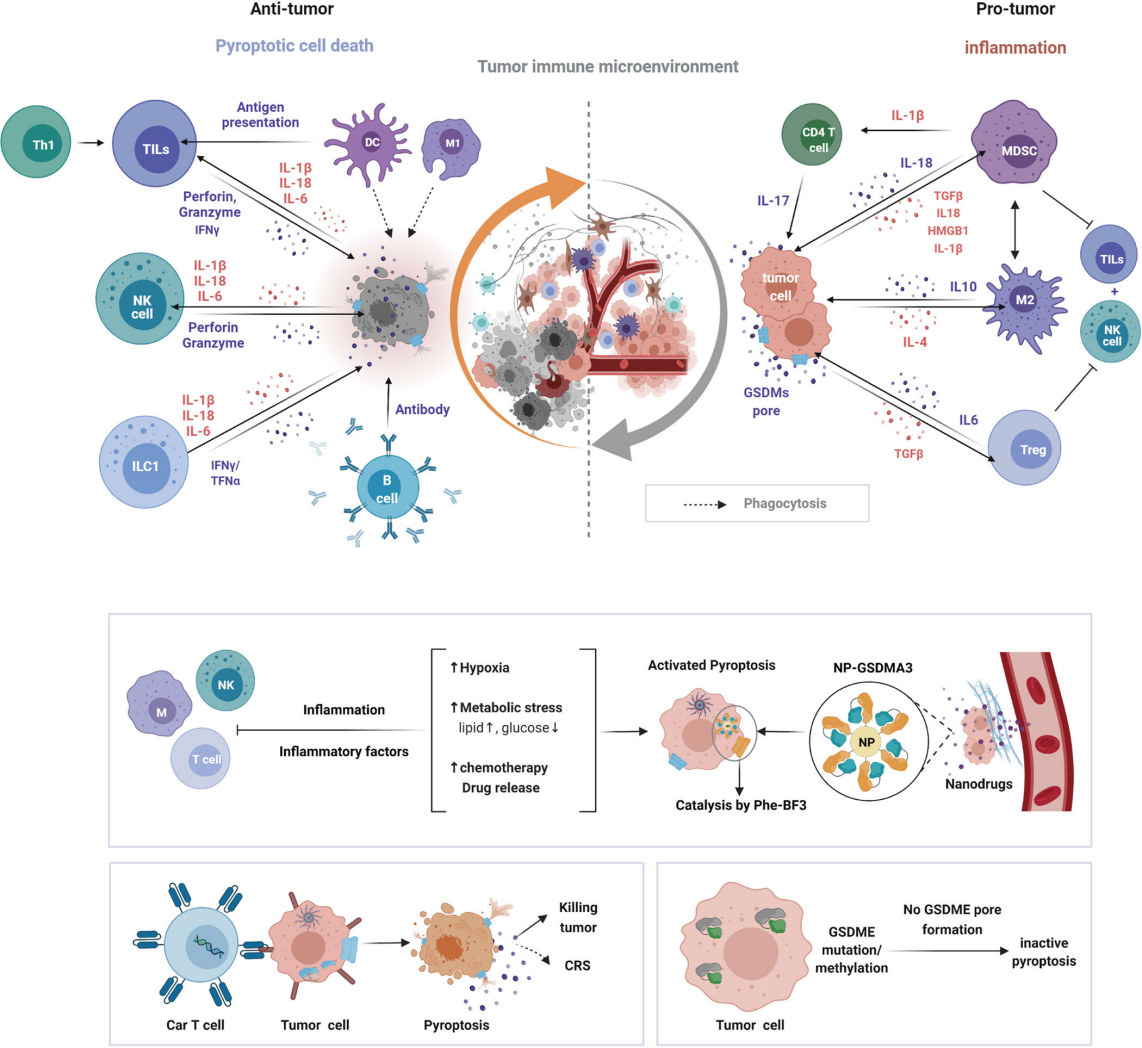
Pyroptosis plays dual roles in the tumor microenvironment. Presented are the dual roles of pyroptosis in the TME. Whether antitumor immunity or pro‐tumor immunity mainly dependent on different inflammatory mediators, time and response levels, as well as tissue types for some GSDMs like GSDME are inhibited in many cancer cells. CAR‐T therapy together with CRS and GSDMA3‐mediated drug delivery are cases for application of pyroptosis. Abbreviations: CRS, cytokine‐release syndrome; ILC1, innate lymphoid cell; M1/M2, macrophage; MDSC, myeloid‐derived suppressor cell; TILs, tumor‐infiltrating lymphocytes

Pyroptosis is a double‐edged sword and plays an important role both in tumorigenesis and antitumor immunity at all stages of tumor development, which shows tumor promoting or tumor suppressive effects in this context, mainly relying on types of tumor, host inflammatory status and immunity, and the involved effector molecules.^12^ Long‐term chronic inflammation in TMEs has a great impact on progression and immunity activity of tumors, representing a major factor in the response of antitumor therapies. As for the double roles of pyroptosis, what can be explained is that, on the one hand, long‐term chronic inflammation could facilitate tumor development, as inflammation caused by pyroptosis facilitates the generation and maintenance of an inflammatory microenvironment surrounding cancer cells. On the other hand, the acute activation of pyroptosis leads to the infiltration of various immune cells, thus repressing tumor development.^248^ In general, the induction of pyroptosis inside tumors might be considered a potential strategy in various cancer treatments.^6^, ^10^, ^45^ (Figure 4)

### 4.1 The antitumor effects of pyroptosis

#### 4.1.1 Inflammatory mediators

The inflammatory state of the TME has implications for the response to immune checkpoint inhibitor (ICI) therapy. By triggering pyroptosis, a form of ICD, converting these immune “cold” tumors to “hot” tumors may alter the TME and the influx of tumor‐infiltrating lymphocytes (TILs). Pyroptotic immune response involves a long process which goes from the release of damage‐associated molecular patterns (DAMPs) (e.g. HMGB1, ATP, etc.) and inflammatory cytokines, directly modulating the innate immune response, to enhancing the recruitment of adaptive immune cells along with increased antigen presentation and TLR activation, leading to broader immune activation.^249^, ^250^ Understanding the complex mechanisms of pyroptosis and how the process alters the TME may have profound effects on future therapeutic strategies in both the up‐front and salvage treatment settings.

Which features of pyroptotic tumor cells contribute to activating antitumor immunity is not currently clear. Inflammatory cytokines and immunostimulatory alarmins released during pyroptosis, including IL‐1β, IL‐18, ATP, and HMGB1, could be prime suspects. During pyroptosis, IL‐1β is activated and released from the GSDM pores to exert an influence on the TME. IL‐1β signaling could induce DC maturation and monocyte differentiation into DCs and inflammatory macrophages. Then, antigens will be represented to T cells to elicit immune killing. In addition, IL‐1β can directly act on cells in the lymphoid compartment to drive antigen‐specific cytotoxic CD8^+^ T cell responses, increase Th1 CD4^+^ T cell numbers, and inhibit the differentiation of immunosuppressive T regulatory cells.^249^, ^250^ Indeed, it has been shown that IL‐1β by intra‐tumoral injections is sufficient to cause CD8^+^ T cell‐mediated tumor regression in models of adenocarcinoma, melanoma, and sarcoma.^251^

IL‐18 can strongly induce γ‐IFN when it combines with IL‐12 or IL‐15, which both upregulate the IL‐18 receptor levels. Thus, IL‐18 exerts an important effect on natural killer (NK) cell recruitment and activation, as well as Th‐1 polarization.^252^

In addition to IL‐1β and IL‐18, other types of cytokines such as IL‐6, are also released from pyroptotic cells. Accumulating evidence shows that IL6 has a profound impact on the adaptive immune response. For instance, IL‐6 signaling can increase T cell immune response to an actively responsive state from a suppressive state, thereby effectively fighting against tumors. Also, IL‐6 is also important for enhancing T cell trafficking to lymph nodes and the tumor sites. Then, they have chances to be activated and further execute their cytotoxic effector functions.^253^ Additionally, IL6 exerts an influence on increasing antibody production, increasing cytotoxic CD8^+^ T cell differentiation and decreasing regulatory T cell differentiation.^253^, ^254^

As a type of alarmins, extracellular ATP reinforces pyroptosis by triggering of purinergic P2 × 7R signaling in infiltrating immune cells or cancer cells.^255^ Like apoptosis, pyroptotic cells could release macrophage attractants, “find‐me” and “eat‐me” signals, the main component of which is ATP. Phagocytosis induction of pyroptotic cells by macrophages using the two signals will ultimately promote CD8^+^ T‐cell activation and IFN‐γ production.^256^ For the positive aspects, the release of HMGB1 from dying tumor cells could force host DCs to process and present tumor antigens. Also, HMGB1 signaling can interact with TLR2 and TLR4 receptors on the surface of neutrophils, monocytes, macrophages, DCs, and lymphocytes, leading to the activation of transcription factors NF‐κB and AP1, the production of inflammatory cytokines such as IL‐6, TNF α, IL‐8, and IL‐1, and an increase in co‐stimulatory molecules required for the cross‐priming of antitumor T lymphocytes.^257^, ^258^

#### 4.1.2 Lymphocyte cytotoxicity

Pyroptosis is essential for lymphocyte cytotoxicity against tumor cells, and pyroptotic tumor cells induce the TME toward an immunostimulatory state. To some extent, a positive feedback loop is formed in anticancer immunity as for the promotion between pyroptosis and cytotoxic lymphocytes. In the case of GSDME, a tumor suppressor which activates pyroptosis, this enhances antitumor immunity.^6^ It is well known that caspase‐3 cleaves GSDME to activate pyroptosis, and the cell killer GZMB has also been reported to activate caspase‐independent pyroptosis in target cells by directly cleaving GSDME (after D270) at the same site as caspase‐3. Indeed, non‐cleavable or non‐pore‐forming GSDME is unable to perform its tumor‐suppressive ability. Interestingly, on the one hand, the antitumor ability of GSDME mainly depends on the killing ability of CD8^+^ T cells, NK cells, granzymes, and perforin. On the other hand, given that immunotherapeutic strategies often aim to improve T cell responses to cancer, the overexpression of GSDME in cancer cells significantly increases the number and function of infiltrating NK cells and antigen‐specific CD8^+^ T lymphocytes inside tumors, as well as the expression of effective molecules including GZMB, perforin, IFN‐γ, and TNF‐α in TILs, together with the phagocytosis of tumor‐associated macrophages (TAMs).^259^, ^260^ GSDME converts more moderate and non‐inflammatory apoptotic death into more rapid and inflammatory pyroptotic death that can stimulate an effective antitumor immune response.^6^ It is also reported that GSDMD participates in cell death mediated by cytotoxic T lymphocytes (CTLs) in lung squamous cell carcinoma and LUAD.^261^

#### 4.1.3 Phagocytosis by macrophages

As for GSDMC, it has been shown that the expression of GSDMC under hypoxia is transcriptionally enhanced by p‐STAT3 and is physically correlated with PD‐L1 and its nuclear translocation. Then, after treatment with TNF‐α derived from macrophages, GSDMC is specifically cleaved by caspase‐8, liberating the NT domain to form pores on the plasma membrane, thereby inducing pyroptosis. The high expression of GSDMCs is also associated with poor patient survival.^46^ Pyroptosis in macrophages induced by sorafenib could trigger NK cell‐mediated cytotoxicity against hepatocellular carcinoma (HCC). Then, pyroptotic macrophages release IL‐18 and CCL5 to enhance the chemotaxis and cytotoxicity of NK cells and eliminate HCC by activating antitumor immunity.^262^

#### 4.1.4 Pyroptosis in other immune cells

In cellular immunity, CTLs and NK cells kill target cells via the delivery of serine protease granzymes by perforin. Notably, lymphocyte‐derived GZMA could cleave GSDMB between CT and NT linker at Lys^244^ site, resulting in pyroptotic cell death in GSDMB‐positive cells killed by NK cells and CTLs.^45^

Tumor cells undergoing pyroptosis release IL‐1β, IL‐18, and various DAMPs, which are signals used to activate and recruit DCs or macrophages to phagocytose the pyroptotic cells and promote their maturation. Then, mature DCs present tumoral antigens to tumor‐specific CTLs, killing pyroptotic tumor cells.^256^, ^263^ Melanoma is known as a “cold” tumor due to the poor responses to ICI therapy; the combination of B‐Raf Proto‐Oncogene, serine/threonine kinase (BRAF) and Methyl Ethyl Ketone (MEK) inhibitors (BRAFi + MEKi) is an FDA‐approved approach to therapy *BRAF V600E/K*‐mutant melanoma. However, the efficacy of the BRAFi + MEKi approach is correlated with immune responses in the TME. Given that pyroptosis could elicit antitumor immunity in the tumor immune microenvironment, the involvement of pyroptosis in ICI treatment is being investigated in new researches. They have found that the treatment of *BRAF*‐mutant melanoma cells with BRAFi + MEKi inhibitors promotes the cleavage of GSDME and the release of HMGB1, all of which are markers of pyroptotic cell death. Interestingly, GSDME‐deficient melanoma represents defective HMGB1 release, the reduced infiltration of tumor‐associated T cells and activated DCs in response to BRAFi + MEKi treatment, and the more frequent tumor regrowth after drug removal. Importantly, BRAFi + MEKi‐resistant diseases show the decreased infiltration of intra‐tumoral T cells and the lack of pyroptosis‐related markers but still retain sensitivity to pyroptosis‐induced chemotherapy.^47^ The data imply that pyroptosis could as an adjunctive therapy to ignite antitumor immune responses for resistant melanoma. In addition, in *KRAS‐*, *EGFR*‐, or *ALK*‐driven lung cancer, upon treatment of diverse small‐molecule inhibitors, robust pyroptotic cell death, associated with GSDME cleavage, and the mitochondrial intrinsic apoptotic pathway are elicited and spread in lung cancer cells uniformly, making concession under genotype‐specific regimens.^264^

#### 4.1.5 New application of pyroptosis in antitumor approaches

As tumor treatment is obstructive and complex, the effect of immunotherapy is gaining increasing prominence. However, the immune checkpoint blockade often responds ineffectively to some tumor treatments as for the low inflammatory response among the TME. Thus, the development of new approaches to boost antitumor immunity is critical. Recently, the Phe‐BF3 desilylation bio‐orthogonal system applied efficiently has been shown to transport desilylation catalyzed by Phe‐BF3 with the NP‐GSDMA3‐mediated delivery into specific mammary tumor cells in mice. The novel technique leads to T cell‐dependent tumor progression along with increasing numbers and the function of CD4^+^, CD8^+^, NK cell, and M1 macrophage populations, while decreasing regulatory T cell, M2 macrophage, monocyte, neutrophil, and myeloid‐derived suppressor cell (MDSC) populations. As a new application, the system uncovers the antitumor immune potential of pyroptosis, suggesting that a GSDM agonist may improve the efficacy of cancer immunotherapy.^10^

Pyroptosis also functions in patients with extrahepatic cholangiocarcinoma (CCA). Tumor‐cell‐derived microvesicles whose plasma membranes are infused with methotrexate could induce pyroptosis in CCA cells through a GSDME‐dependent pathway. Then, intracellular contents released from pyroptotic CCA cells activate patient‐derived macrophages to produce pro‐inflammatory cytokines, which attract the secondary accumulation of neutrophils to the tumor site. The procedure is relevant to the upregulation of uridine diphosphate glucose and complement C5, leading to degradation of the stromal barrier in the CCA TME. The approach has been proved to relieve biliary obstruction in nearly 25% of CCA patients.^265^

Another application of pyroptosis is that of a commonly used tumor‐targeting nanoliposome loaded with cisplatin, which is applied in drug delivery and administration. Combining decitabine (DAC) with chemotherapy nanodrugs to manage drugs to activate and upregulate the caspase‐3/GSDME signal, further triggers pyroptosis in tumor cells, therefore strengthening the immunological effect of chemotherapy with cisplatin in a mouse triple‐negative breast cancer model. Also, DAC can demethylate the GSDME gene in tumor cells with specific tumor‐bearing mice.^266^

The chimeric co‐stimulatory converting receptor is designed to disturb the PD‐1 pathway which enhances the activity of chimeric antigen receptor (CAR)‐NK cells to fight solid tumors. Then, the antitumor activity of NK92 cells was enhanced by the neo‐complex PD1‐NKG2D‐41BB receptor, which is mainly dependent on pyroptosis activation.^267^

### 4.2 The pro‐tumor effects of pyroptosis

In certain contexts, ICD caused by pyroptosis may also be pro‐tumorigenic. This is consistent with the complicated roles of inflammation in both enhancing and inhibiting tumor growth, thus suggesting that the timing, levels, and components of pyroptosis induction need to be closely controlled. It is MDSCs which are substantial contributors to the immunosuppressive TME and are closely associated with tumor progression.

The main alarmin, HMGB1, released by pyroptotic cells, could promote tumor cell survival, which largely suggests that HMGB1 drives the accumulation of MDSCs by inducing autophagy, thereby maintaining MDSC viability.^268^, ^269^, ^270^ In addition, HMGB1 also enhances the immune suppression of MDSCs by promoting their differentiation from bone marrow progenitor cells and the suppressive activity on antigen‐driven activation of CD4^+^ and CD8^+^ T cells.^271^ Moreover, HMGB1 also increases MDSC‐mediated IL‐10 production, enhancing crosstalk between MDSCs and macrophages, and down‐regulating L‐selection on T cells to perturb their location to lymph nodes.^271^

Besides, IL‐18 exerts antitumor activity when it combines with other inflammatory cytokines, paradoxically, IL‐18 can promote Th2 responses and angiogenesis, which leads to increased migration and invasion in tumors in the absence of other cytokines.^272^ Thus, IL‐18 may have a differential impact on tumor progression, which partly depends on the makeup of cytokine types. In addition to the main contents of pyroptotic production, intrinsically, another member, IL‐6, is found to play a role in tumor cells by supporting malignant progression including proliferation, metastatic dissemination, and survival by connecting with numerous downstream mediators. Furthermore, IL‐6 can exert an impact on other cells within the TME to maintain a favorable growth ambience of tumor cells for the ease of angiogenesis and tumor evasion from immune surveillance. In turn, tumor cells can also express or respond to IL‐6, leading to increased proliferation and invasion.^253^

Moreover, TAMs also contribute to the immunosuppressive microenvironment, which display an upregulated NLRP3 inflammasome activity, thereby significantly promoting the expression of IL‐1β and thus aggravating inflammation and favoring the progression of CRC.^12^

## 5 CONCLUSION

Cell death is a complex regulatory net which decides cell fate. Among the forms of programed cell death, pyroptosis and necroptosis all are ICD, while apoptosis is non‐inflammatory. In some contexts, they all contribute to the homeostasis of the host by different molecular pathways. However, given that (1) caspase‐8 involves both the pyroptosis, necroptosis and apoptosis pathways^273^, ^274^; (2) activation of the NLRP3 inflammasome can trigger both pyroptosis and necroptosis^273^; (3) GSDME‐mediated pore formation on the mitochondrial membrane is favorable for the release of cytochrome *c* and activation of apoptosome, augmenting apoptosis,^97^ it has been reported that pyroptosis induced by TNF‐α+CHX and navitoclax in cancer cells could function through the BAK/BAX‐caspase‐3‐GSDME signaling pathway.^178^ Thus, the crosstalk between the three types of programmed cell death suggests that they do not function independently and connect, even affecting each other under certain conditions. Although they could be distinguished by the cell nucleus, cytoplasmic membrane, morphological characteristics and cell staining, the detailed regulatory mechanism and crosstalk require more attention (Table 3).

**TABLE 3:** Comparison between types of programmed cell death (pyroptosis, necroptosis, and apoptosis)

Subject	Characteristics	Inflammatory cell death		Non‐inflammatory cell death
		pyroptosis	necroptosis	apoptosis
Properties	Programmed cell death	+	+	+
	Inflammation	+	+	–
Nucleus	DNA damage	+	+	+
	Chromatin condensation	+	+	+
	Intact nucleus	+	+	–
Plasma membrane	Membrane blebbing	+	+	+
	Pore formation	+	+	–
	PS exposure	+	+	+
	Osmotic lysis	+	+	–
	Membrane integrity	–	–	+
	Cell shrink	–	–	+
	Cell swelling	+	+	–
Key mediators	Gasdermin cleavage	+	–	–
	PARP cleavage	–	–	+
	MLKL cleavage	–	+	–
	Inflammasomes	+/‐	+/‐	–
	Caspase‐1 activation	+	+	–
	Caspase‐2 activation	–	–	+
	Caspase‐3 activation	+	–	+
	Caspase‐4 activation	+	–	–
	Caspase‐5 activation	+	–	–
	Caspase‐6 activation	+	–	+
	Caspase‐7 activation	–	–	+
	Caspase‐8 activation	+	+	+
	Caspase‐9 activation	+	–	+
	Caspase‐10 activation	–	–	+
	Caspase‐11 activation	+	–	–
Cell staining	Annexin V staining	+	+	+
	PI staining	+	+	–
	TUNEL staining	+	+	+
	7‐AAD staining	+	+	–
Membrane repair	ESCRT system	+	+	–

*Note*: −, absent; +, present.

**FIGURE 5:**
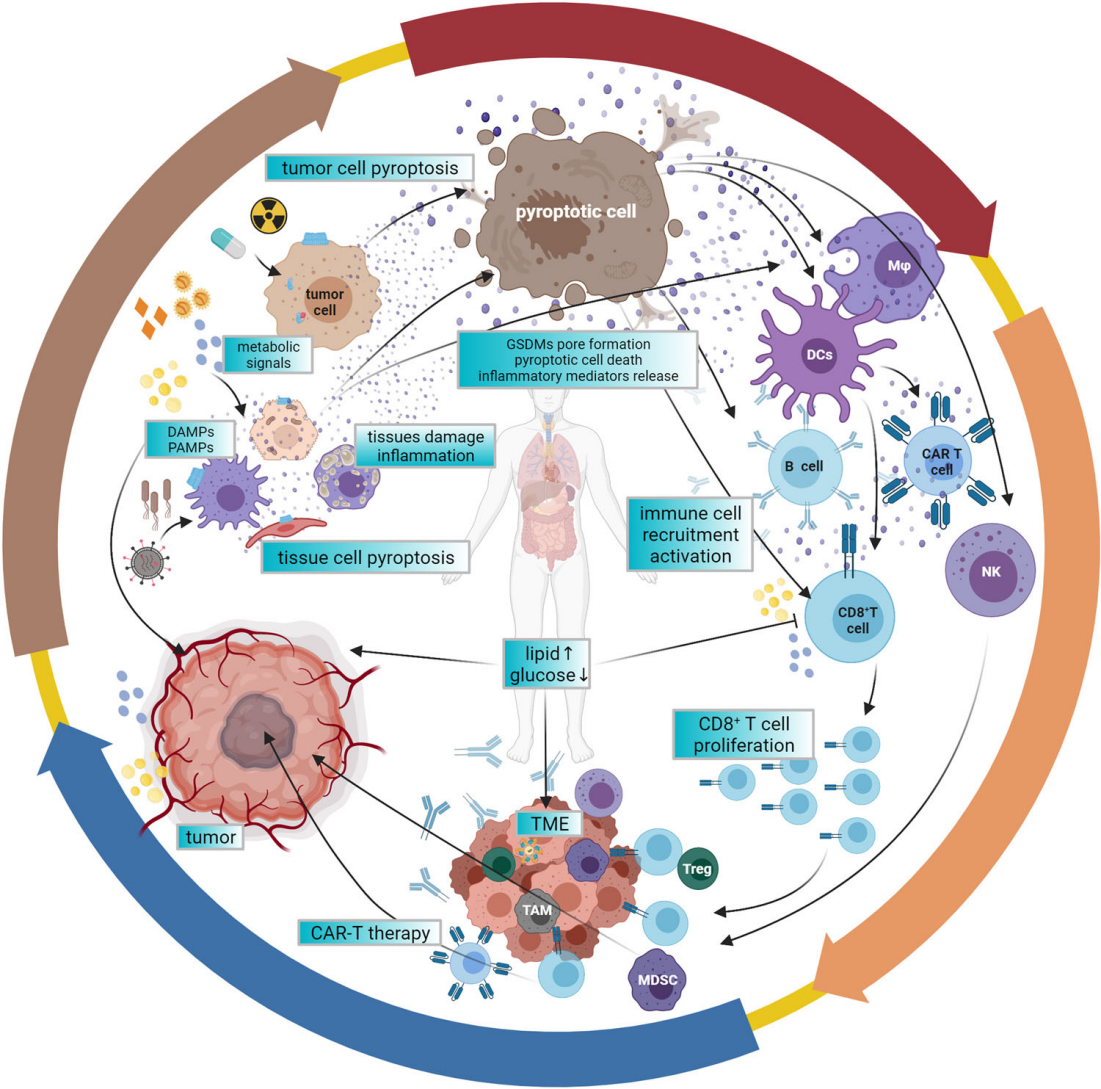
The network between pyroptosis, metabolic signals, and tumor immune microenvironment. Pyroptosis can be induced by different metabolic signals and killer immune cells attach. In turn, inflammatory mediators released by pyroptotic cells can regulate the numbers and function of immune cells, as well as metabolic conditions. Besides, metabolic molecules such as glucose and lipids can also mediate the balance between immune cells and tumor cells. In total, pyroptosis, metabolic signals, and tumor immune microenvironment form a complex regulatory network. Abbreviation: TME, tumor microenvironment.

Metabolic nutrients play a central role in monitoring the immune cell fate, and tumors, especially solid tumors, likely produce an immunosuppressive TME which is deprived of metabolic nutrients including glucose and free fatty acids; in contrast, this is rich in metabolic wastes, such as hypoxic and lactic acid.^275^, ^276^ A feature of TME is the more favorable of rapid proliferation of tumors, since tumors could increase metabolic substrate flexibility and resist mitochondrial oxidative metabolism, just like the Warburg effect.^277^, ^278^ However, the metabolic demands of tumors lead to severe dysfunction and abnormal differentiation in cytotoxic immune cells, including T cells and NK cells, which principally use glucose by glycolysis and mitochondrial oxidative phosphorylation.^278^, ^279^, ^280^, ^281^, ^282^ Besides, elevated lipid metabolism and glucose/amino acid dearth also increase the production of reactive ROS and suppressive epigenetic modifications in T cells.^280^, ^283^ The metabolic characteristics of TME could further damage antitumor immunity and accelerate malignant progression. Moreover, obesity and other diseases caused by metabolism dysregulation are accompanied by inflammation to different degrees, which may induce a tendency toward tumor development if the inflammation exists for a long time.^284^, ^285^, ^286^ Thus, on the one hand, fatty acids, HG levels, and other metabolic signals could activate inflammasomes and pyroptosis, producing various inflammatory factors.^192^, ^197^, ^199^, ^287^ On the other hand, the metabolism statue also regulates the battle between immune cells and tumor cells^288^ (Figure 5).

The success of immunotherapy is largely dependent on the high activity of intratumoral CD8^+^ T cells, and the therapeutic effect has been shown in some solid tumors such as melanoma and lung cancer.^47^, ^289^, ^290^, ^291^, ^292^ However, the ineffective ICI is in part caused by the absence or poor response of effector immune cells.^47^, ^289^, ^293^ To some extent, cytokines, chemokines, and other inflammatory factors, which could recruit immune cells including toxic T cells, NK cells, and TAMs, may also contribute to the effect of immunotherapy. Thus, ICIs may be more effective if combined with other therapies that can improve the number and function of infiltrating T cells, NK cells, and inflammatory macrophages.^10^, ^265^, ^266^ Interestingly, preclinical models have suggested that ICD‐inducing therapy through pyroptosis could recruit CD8^+^ T cells and other lymphocytes as well as myeloid cells to the surrounding tumor lesions in the TME.^47^ In the case of melanomas, inducing pyroptosis with the DNA topoisomerase inhibitor etoposide could reduce tumor growth in BRAF inhibitor‐ and MEK inhibitor‐resistant melanomas.^47^

However, ICIs, like CAR‐T cell therapy, have also been reported to induce cytokine‐release syndrome (CRS), which may counteract the effectiveness of CAR‐T cell therapy partly as a severe consequence of extensive pyroptosis. It is shown that CAR‐T cells could rapidly activate caspase‐3 in target cells through the release of GZMB. Then, the latter cleaves GSDME, causing extensive pyroptosis. Consequently, other pyroptotic factors are triggered and further activate the caspase‐1/GSDMD pathway in macrophages, which results in the increased release of cytokines and more serious CRS.^294^ Thus, the combination of ICIs and pyroptosis‐inducing therapies may further increase the risk.^295^, ^296^

Fortunately, the research has shown that nanodrugs potentially reduce the side effect of ICIs, as the application of nanoparticles could promote the more appropriate distribution of pyroptosis inducers in target cells other than the adjacent tissues. Thus, the new utilization of pyroptosis will improve therapeutic effects.^10^, ^297^, ^298^

Although the molecular features of GSDM family members, especially GSDMD, are gradually revealed in pyroptosis, the exact regulatory mechanisms of other GSDMs are not very clear. In addition, large inflammatory factors are found to be released from immune cells but not cancer cells, although they exert an important impact on the latter. Also, they have the opposite impact in different regions and evolving periods of TME. In summary, more in‐depth and innovative exploration into the roles of pyroptosis in metabolism and the TME are required in the future.

## AUTHOR CONTRIBUTIONS

Lutao Du, Chuanxin Wang, and Juan Li designed the manuscript. Tiantian Du, Jie Gao, and Peilong Li searched the literature and wrote the manuscript. Lutao Du, Chuanxin Wang, Juan Li, Jie Gao, and Peilong Li provided the funding. Xiaoyan Liu, Qiuchen Qi, and Yunshan Wang provided the conception, edited, and polished the manuscript. All authors read and approved the final manuscript.

## CONFLICT OF INTEREST

The authors declare that they have no conflict of interest.
